# Cognitive skills and reading in adults with Usher syndrome type 2

**DOI:** 10.3389/fpsyg.2015.00326

**Published:** 2015-03-25

**Authors:** Cecilia Henricson, Björn Lidestam, Björn Lyxell, Claes Möller

**Affiliations:** ^1^Swedish Institute for Disability Research (SIDR)Linköping, Sweden; ^2^Linnaeus Centre for Research on Hearing and Deafness (HEAD)Linköping, Sweden; ^3^Department of Behavioral Sciences and Learning, Linköping UniversityLinköping, Sweden; ^4^Audiological Research Centre, Örebro University HospitalÖrebro, Sweden; ^5^School of Medicine and Health, Örebro UniversityÖrebro, Sweden

**Keywords:** deafblindness, Usher syndrome, phonological skill, lexical skill, working memory, reading

## Abstract

**Objective:** To investigate working memory (WM), phonological skills, lexical skills, and reading comprehension in adults with Usher syndrome type 2 (USH2).

**Design:** The participants performed tests of phonological processing, lexical access, WM, and reading comprehension. The design of the test situation and tests was specifically considered for use with persons with low vision in combination with hearing impairment. The performance of the group with USH2 on the different cognitive measures was compared to that of a matched control group with normal hearing and vision (NVH).

**Study Sample:** Thirteen participants with USH2 aged 21–60 years and a control group of 10 individuals with NVH, matched on age and level of education.

**Results:** The group with USH2 displayed significantly lower performance on tests of phonological processing, and on measures requiring both fast visual judgment and phonological processing. There was a larger variation in performance among the individuals with USH2 than in the matched control group.

**Conclusion:** The performance of the group with USH2 indicated similar problems with phonological processing skills and phonological WM as in individuals with long-term hearing loss. The group with USH2 also had significantly longer reaction times, indicating that processing of visual stimuli is difficult due to the visual impairment. These findings point toward the difficulties in accessing information that persons with USH2 experience, and could be part of the explanation of why individuals with USH2 report high levels of fatigue and feelings of stress (Wahlqvist et al., 2013).

## Introduction

Impairment in both hearing and vision, deafblindness, causes major reduction in intake of sensory information from the environment. There can be several etiologies behind deafblindness, but Usher syndrome is one of the most common causes (Pennings, 2004; Sadeghi, 2005). The clinically estimated prevalence of Usher syndrome is reported to be 2.4–6.2 individuals out of 100 000 people (Sadeghi et al., 2004b). The prevalence is similar worldwide, though which type of the syndrome that is most common differs locally (Sadeghi et al., 2004b). In Sweden the clinically defined types 1 and 2 of the syndrome are most common, and type 3 is unusual (Sadeghi et al., 2004b). In the present study, focus is on adults with USH2. Individuals with USH2 have a congenital moderate-to-severe hearing loss, and the hearing loss remains relatively stable over the lifespan (Sadeghi et al., 2004a). Individuals with USH2 have a severely limited, central visual field and suffer visual deficts such as poor photo- and contrast sensitivity due to the retinal disorder retinitis pigmentosa (RP; Sadeghi et al., 2004b; van Wijk et al., 2004). The first symptoms (poor contrast sensitivity and night blindness) of RP, are typically evident during ages 5–10 years but is commonly diagnosed in the late teens in individuals with USH2 (Sadeghi et al., 2004b). The degenerative process in the retina typically stabilizes at about 40–50 years of age (Sadeghi et al., 2004b).

Several of the genes causing Usher syndrome have been mapped and described, and the medical aspects of Usher syndrome have also received much attention in research, however, the cognitive functioning of individuals with the syndrome has not been in focus. In the present study three basic cognitive skills were examined in adults with USH2; WM, phonological and lexical skills. Several studies have demonstrated that the capacity of, and efficient interplay between, these cognitive skills are highly important to the amount of understanding achieved when decoding language, whether in speech (Rönnberg et al., 2010), or written form (Engel de Abreu et al., 2011). The decoding of information in speech relies partly on phonological skills (Rönnberg et al., 2010; Classon et al., 2013). The separate words have to be identified in the continuous speech signal, and identification is mediated by matching the phonological sequences to phonological representations stored in long term memory. In this way, the burden on the storage component of phonological WM is reduced, and more resources can be directed to processing the semantic content (Rönnberg et al., 2010). A number of studies have shown that hearing impairment (HI), both congenital and non-congenital in nature, is associated with reduced efficiency of phonological processing, especially less stable phonological representations and reduced phonological WM (Lyxell et al., 1996, 1998, 2009; Andersson, 2001; Spencer and Tomblin, 2009; Henricson et al., 2012; Lazard et al., 2012; Classon et al., 2013). Several studies using the Reading Span test have found that individuals with long term hearing loss display lower results on the test (Lyxell et al., 1996; Rönnberg et al., 2010), which also suggests a decrease in WM for verbal materials. These findings should be highly relevant also in the case of the group with USH2, but whether they apply to the same extent has not been investigated.

A better understanding of the cognitive functioning of the group with USH2 is at the foundation of developing better assistance and rehabilitation, which could increase the well-being for individuals. Information on the group’s performance on cognitive measures could also offer insights on the influence of perceptual information from the auditory and visual senses on cognitive performance. As mentioned previously, the cognitive skills that are examined in the present study are at the basis of other complex abilities, and could be of specific importance to reading, for example. In normal hearing (NH) individuals the correlation between phonological skills and reading skills is typically most pronounced in the initial stages of learning to read (Lundberg, 2009; Schaffner and Schiefele, 2013). Children with cochlear implants (CIs) constitute a group who often display low phonological skills (Lyxell et al., 2009; Geers and Sedey, 2011; Dillon et al., 2012), and for example Geers et al. (2008) have shown that despite reading at level with children with NH at the ages 10–12 years, many children with CI display low performance on tests of reading comprehension in late adolescence. A possible explanation could be that since relying on phonological skills is effortful for many children with CI, the alternative is to use the salient visual cues, such as shapes of words and letters when decoding text. It seems probable that children with CI apply an orthographic reading strategy (Lyxell et al., 2009; Geers and Sedey, 2011), which might not be sufficient in order to reach full comprehension of complex texts. Because of the congenital hearing loss, the development of phonological and lexical skills is at risk in individuals with USH2. Also, the progressive visual loss could be interfering with the retrieval of information such as the mentioned salient visual cues of text, but also with the individual’s ability to learn and use lip-reading (a skill which relies on an understanding of spoken language phonology, and hence could maintain and refine the phonological skills), further complicating the development of language skills. Since the development of reading skills (e.g., decoding and comprehension) depends on phonological and lexical abilities in individuals with NH, the present study aimed to examine this relationship in individuals with USH2. More specifically, the present study investigated phonological and lexical skills, WM and reading comprehension in individuals with USH2 compared to matched controls with NHV.

## Materials and Methods

### Participants

The group of participants consisted of 13 individuals (3 women, 10 men) with USH2 in the ages 21–60 years (*M* = 38.8, SD = 12.7 years, see **Table 1**). The participants’ ages were distributed such that four individuals were between 20 and 30 years, two between 31 and 40 years, five between 41 and 50 years, and two individuals between 51 and 60 years of age. All were recruited through the Örebro Audiological Research Centre’s national database on Usher syndrome, in which they had been entered after receiving their diagnosis of USH2a, as results of clinical and genetic investigation. All participants with USH2 had a symmetric, sensorineural, sloping hearing loss which was moderate to severe (Pure Tone Average over four frequencies (PTA4) left ear, *M* = 66.2 dB, SD = 11.6 dB; PTA4 right ear, *M* = 67.5 dB, SD = 13.3 dB, see **Table 1**). Speech discrimination in noise (signal/noise + 4 dB) was in all participants with USH2 between 50% and 60% correctly identified words, which due to the hearing loss was an expected level of performance. Information on participants’ visual field was retrieved from the Örebro Audiological Research Centre’s database on Usher syndrome and is reported as the calibrated Goldmann hemispheres. The Goldmann hemispheres categorizes loss of visual field into five phenotypes where 1 = normal visual field, 2 = presence of a partial or complete ring scotoma, the latter either extending or not extending into periphery, 3 = concentric central field loss with a remaining peripheral island, less than one-half of the field circumference, 4 = marked concentric loss (visual field of less than, or equal to, 10^∘^), and 5 = no visual field (blindness; Sadeghi et al., 2004b). The classification of participants’ visual fields is displayed in **Table 1**, as is data on participants’ visual acuity. Visual acuity is reported in the decimal scale, where a value of 1–0.6 is considered normal vision, and 0.05 indicates functional blindness. All participants had Swedish as their primary language. All of the participants with USH2 had completed the Swedish comprehensive school of 9 years, and the Swedish upper secondary school of 3 years. Seven of the participants with USH2 had studied for one up to 5 years of university education, and six had vocational educations.

**Table 1:** **Data on age, hearing thresholds (PTA4) and vision for the participants**.

	USH2, *M* (SD)	Control, *M* (SD)
Age, years	38.8 (12.7)	38.4 (11.0)
PTA4, dB, Left ear	66.2 (11.6)	3.7 (5.1)
PTA4, dB, Right ear	67.5 (13.3)	3.3 (3.6)
Visual field, left *Goldman Hemispheres*	3 (1.2) minimum–maximum: 1–5	Not applicable
Visual field, right *Goldman hemispheres*	3 (1.1) minimum–maximum: 1–4	Not applicable
Visual acuity, left *Decimal Scale*	0.47 (0.37) minimum–maximum: 1–0.05	Not applicable
Visual acuity, right *Decimal Scale*	0.41 (0.35) minimum–maximum: 1–0.05	Not applicable

*Visual field reported according to the Goldman standard (Sadeghi, 2005), where a classification of 1 = normal visual field and 5 = no visual field. Visual acuity reported in the decimal scale, where 1 = normal acuity and 0.05 = functional blindness. M, mean value of the group, SD, standard deviation.*

A control group of 10 persons (four women, six men) in the ages 23–60 years (*M*= 38.4, SD = 11.0), with NH and normal or corrected-to-normal vision was selected to match the group with USH2 with respect to age and educational level. Audiograms were measured on all participants (PTA4 left ear, *M* = 3.7 dB, SD = 5.1 dB; PTA4 right ear, *M* = 3.3 dB, SD = 3.6 dB, see **Table 1**), and vision was reported by each participant to be normal when using corrections such as glasses or lenses. None reported using any other visual facilitation in their every-day life. All of the participants in the control group with NHV had completed the Swedish comprehensive school of 9 years, and the Swedish upper secondary school of 3 years. Six of the participants with NHV had studied for one up to 5 years of university education, and four had vocational educations.

Prior to their participation, all participants received letters of information describing the study aims, methods, and on how data would be reported. All participants provided written consent.

### Cognitive Tests

The test session lasted for 2–2.5 h and included tests of WM, phonological skill, lexical access, phonological WM, and reading comprehension. The tests were given in a set order, but half of all participants were given the tests in reversed order to balance potential order effects. Six of the tests were presented visually (text), and one test was presented auditorily. The six tests which contained visual stimuli (text) were displayed on a computer screen (Dell, LCD, 22^′′^). Color settings for contrast and font sizes 16, 24, 26, 32, 36, 42, 50, 70, and 90 points and could be specified by each participant to enhance visibility and accommodate for the varying degree of visual problems in each participant with USH2. None of the participants with USH2 chose a font size smaller than 24 or greater than 42 points. All participants with USH2 preferred the setting with yellow text on black background, which is the option with highest degree of visual contrast. All participants in the control group also took the tests in this high contrast setting. The stimuli in Serial Recall of Non-words was presented auditorily. Before the test session all participants in the group with USH2 had their hearing aids checked, to ensure that the devices were functioning correctly. At the session all participants had access to further technology, such as tele-coil, loudspeakers, and FM-systems; radio communication units specifically designed for hearing aid reinforcement, were also available. Ten of the participants chose to use the FM-system at some point during, or through the whole of the test session. A sample sentence, not included in the actual test, was used to set the sound level to a comfortable loudness for the participant, before starting the test with the recorded voice. Each experimenter ensured that their voice could be perceived clearly, so that instructions could be heard without problem, before starting the session.

Regarding the control group participants with NHV, the recorded voice was presented through loudspeakers (Logitech S-100), which the participant set to a comfortable level of loudness while listening to the sample sentence. The loudspeakers were positioned on either side of the computer screen, directly in front of the participants. Each experimenter made sure that listening conditions were as good as possible for the control group participants during the test session.

### Verbal Ability: Antonyms

This test has previously been used in Lyxell et al. (1996). It was presented in text on screen. The task was to identify the pair of words which were each other’s antonyms in a set of five words. The participant had 5 min to complete as many items as possible. Performance was scored as number of correctly identified pairs of antonyms, of a maximum of 29 items.

### Speed of Visual Judgment: Physical Matching

This test has previously been used in Lyxell et al. (1996). It was presented in text on screen. The task was to identify whether a displayed pair of letters were identical or different. For the identical condition to be valid, both letters have to be the same, and they have to be either in upper or lower case (e.g., “e – e”). Each item was presented for 2 s with 1 s between tasks, and total number of items was 16. Performance was scored as percentage correct judgments, and mean reaction time (RT) for correct answers was recorded.

### Lexical Access: Lexical Matching

This test has previously been used in Lyxell et al. (1996). Single syllable words or non-words were presented, one at a time, on screen. The task was to judge whether the displayed word was a real word or not and push a button accordingly. There were 40 items, each displayed for a maximum of 5 s with 1 s intervals between items. Performance was scored as percentage correct judgments, and mean RT for correct answers was recorded.

### Phonological Processing: Rhyme Judgment

This test has previously been used in Lyxell et al. (1996), and Classon et al. (2013). The test items were presented in text on screen, and the task was to judge whether pairs of words rhyme or not and push a button accordingly. The participant was instructed to disregard spelling and lettering of the words and focus on their sound (e.g., “MUSTASCH – pistage” makes a rhyme in Swedish). The total number of items was 32. Each item was presented for a maximum of 5 s, with 1 s interval between items. Performance was scored as percentage correct judgments, and mean RT for correct answers was recorded.

### Complex Working Memory: Reading Span

This test has previously been used in Lyxell et al. (1996), Classon et al. (2013), and Ng et al. (2013). This test was presented in text on screen. The participant was presented with sequences of sentences consisting of three words. The first sequence consisted of three sentences, with a maximum of five sentences in one sequence. There were two trials at each level. The sentences were presented word by word, and after each sentence the participant had to judge whether the content was semantically anomalous or not (e.g., “Pots jump high” or “Bikes have wheels”). After a sequence was complete, the task was to repeat either the first, or last, word of each sentence. The participant did not know in advance whether the task would be to repeat the first or last words. The total number of sentences was 24. Each word in each sentence was displayed for 0.8 s with an interval of 0.75 s between them. The interval between sentences was 2 s, during which the participant replied to whether the sentence was absurd or not. Performance was scored as percentage of correct words recalled in a free-recall criterion.

### Phonological Working Memory: Serial Recall of Non-Words

Before starting this test, all participants listened to a sample of the recorded voice in order to set sound to a comfortable and audible level. The task was to repeat sequences of one syllable non-words, all with consonant-vowel-consonant structure. The sequence length started at two words, increasing with one word after three trials at each level, up to a maximum of seven words in sequence. The test was terminated if the participant failed to repeat the correct number of items in a sequence on two attempts. The total number of words was 81, with a total of 162 consonants. Performance was scored in two ways: (1) p.corr.c of recalled words, and (2) Longest recalled sequence.

### Reading Comprehension: Gates MacGinitie

This test was presented in text on screen. Short passages of text on different subjects are presented. The task was to read through each passage and answer multiple choice questions about the contents, or implications, of the text. Performance was scored as number of correct answers of maximum 42 answers.

### Statistics

The data were analyzed for group differences using the Mann–Whitney *U*-tests, with a significance level set to *p* < 0.05. In cases where participants were unable to perform a test, the person was excluded from analyses (i.e., for Reading Span there were two missing values, and analysis was run on eleven subjects). Effect sizes are presented as Pearson *r* values. Since there is a wide age range among participants, Spearman’s correlations were also performed to examine the impact of age on performance. Spearman’s correlations are also used to examine the impact of visual status and degree of hearing loss on performance.

## Results

### Verbal Ability: Antonyms

There was no significant difference between the groups on this test, *U*= 94.50, *z* = 1.84, *p* = 0.07, *r* = 0.38 (USH2: *M* = 14.9 and SD = 4.4; NHV: *M* = 18.3 and SD = 3.6; See **Table 2** for details on performance in the groups). However, the variation in performance was higher in the group with USH2, with three individuals performing above the mean rank value of the control group (15), and six below.

### Speed of Visual Judgment: Physical Matching

**FIGURE 1:**
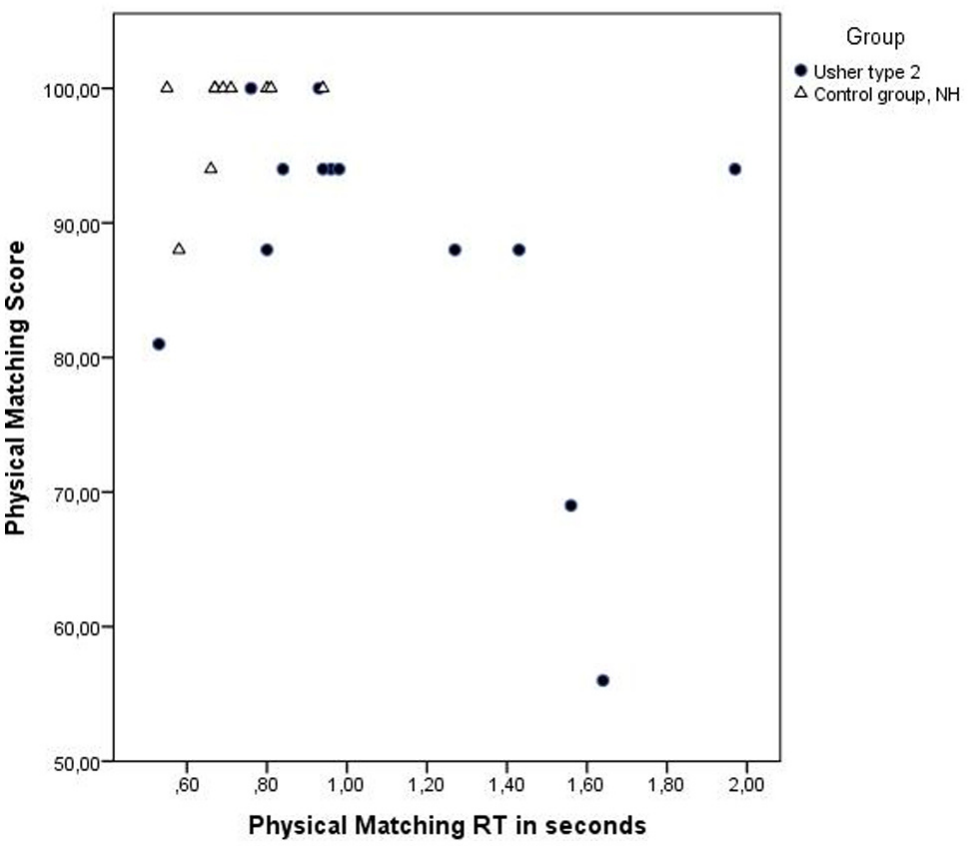
**Displaying reaction time (RT) in seconds and score (% correct answers) for each individual on the test Physical Matching.** Individuals with USH2 are displayed as filled circles and individuals in the control group as triangles. The difference in performance among individuals with USH2 is greater than in the control group with NVH. Performance on this test was affected by degree of visual impairment.

The control group had significantly higher scores on this test, *U*= 109.00, *z* = 2.88, *p* = 0.04, *r* = 0.60 (USH2: *M* = 87.6 and SD = 12.6; NHV: *M* = 98.2 and SD = 4.0), and also had significantly shorter RTs, *U*= 18.00, *z* = 2.92, *p* = 0.04, *r* = 0.61 (USH2: *M* = 1.1 and SD = 0.4; NHV: *M* = 0.7 and SD = 0.1; See **Table 2**, and **Figure 1**, for details on performance in the groups). There were seven participants in the group with USH2 who performed between 94 and 100%, and six with performance below 94%, whereas in the control group only one participant performed below this score. Regarding RTs, 12 participants with USH2 had RTs longer than 0.7 s, compared to only three participants in the control group.

**Table 2:** **Median, range, minimum–maximum, mean value (M), and standard deviation (SD) of performance on the tests in the two groups**.

		Verbal ability	Reading span percent	Physical matching score	Physical matching RT	Lexical matching score	Lexical matching RT	Rhyme judgment score	Rhyme judgment RT	Reading comp. GatesMG	SRN p.corr.c.	SRN longest sequence
**USH2**
N	Valid	13	11	13	13	13	13	13	13	9	13	13
	Missing	0	2	0	0	0	0	0	0	4	0	0
Median		15.0	58.3	94.0	0.96	95.0	1.08	72.0	2.01	43.0	37.5	4.0
Range		15.0	38.2	44.0	1.44	38.0	2.27	56.0	3.09	24.0	36.4	3.0
Minimum		10.0	36.8	56.0	0.53	62.0	0.61	44.0	1.04	24.0	28.4	3.0
Maximum		25.0	75.0	100.0	1.97	100.0	2.88	100.0	4.13	48.0	68.4	6.0
*M*		14.9	54.6	87.7	1.1	92.5	1.4	74.6	2.1	39.0	42.1	3.9
SD		4.4	12.8	12.6	0.4	10.1	0.7	18.2	1.0	8.5	11.9	0.9
**NHV**
N	Valid	10	10	10	10	10	10	10	10	10	10	10
	Missing	0	0	0	0	0	0	0	0	0	0	0
Median		19.0	66.7	100.0	0.68	98.0	0.78	98.5	1.16	44.5	54.0	5.0
Range		10.0	54.2	12.0	0.39	12.0	1.03	25.0	1.43	7.0	40.8	3.0
Minimum		13.0	45.8	88.0	0.55	88.0	0.64	75.0	0.98	40.0	36.4	4.0
Maximum		23.0	100.0	100.0	0.94	100.0	1.67	100.0	2.41	47.0	77.2	7.0
*M*		18.3	69.6	98.2	0.7	96.4	0.9	95.7	1.3	44.4	56.7	4.9
SD		3.6	14.7	4.0	0.1	4.2	0.3	7.8	0.5	2.2	12.5	1.0

### Lexical Access: Lexical Matching (see **Figure 2**)

**FIGURE 2:**
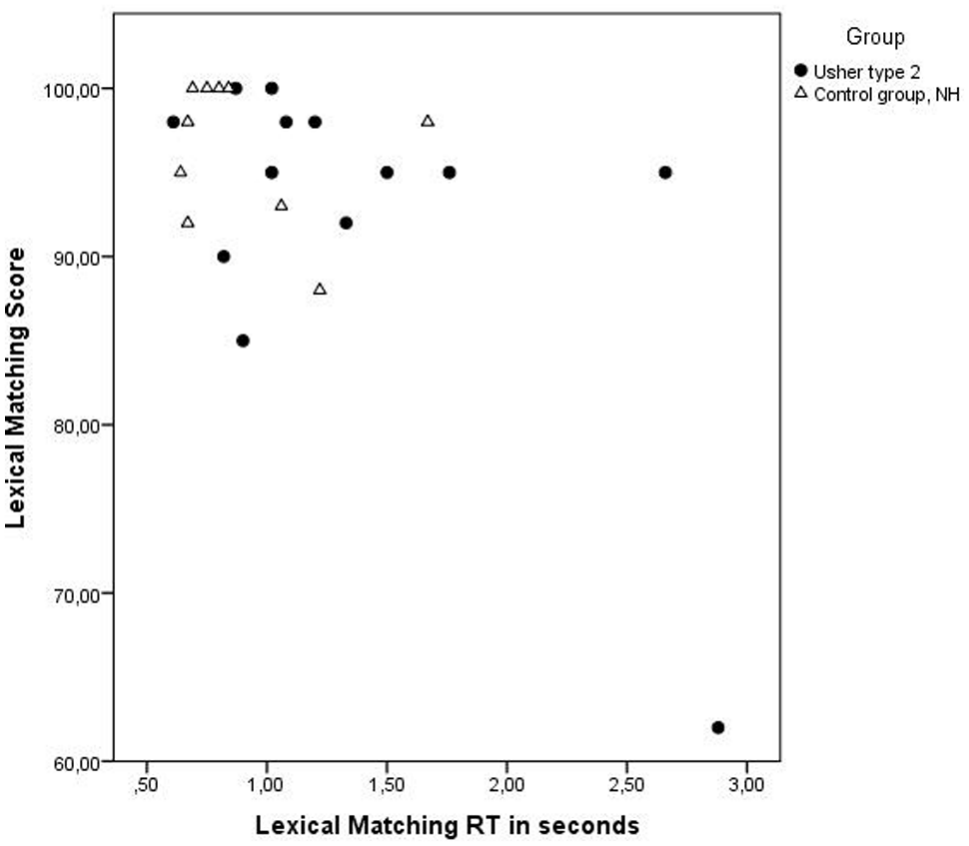
**Displaying RT in seconds and score (% correct answers) for each individual on the test Lexical Matching.** Individuals with USH2 are displayed as filled circles and individuals in the control group as triangles. The difference in performance among individuals with USH2 is greater than in the control group with NVH. Performance on this test was affected by degree of visual impairment.

There was no significant difference in performance between groups regarding score, *U*= 82,50, *z* = 1.11, *p* = 0.27 (USH2: *M* = 92.5 and SD = 10.1; NHV: *M* = 96.4 and SD = 4.2), but there was a significant difference in RT, *U*= 32.00, *z* = 2.05, *p* = 0.04, *r* = 0.43 (USH2: *M* = 1.4 and SD = 0.7; NHV: *M* = 0.9 and SD = 0.3) on this test (See **Table 2**, and **Figure 2**, for details on performance in the groups). Two participants with USH2 and one participant in the control group performed below 90% correct. All except one participant with USH2 had an RT longer than 0.8 s, in comparison to the control group where only three had RTs longer than 0.8 s.

### Phonological Processing: Rhyme Judgment

**FIGURE 3:**
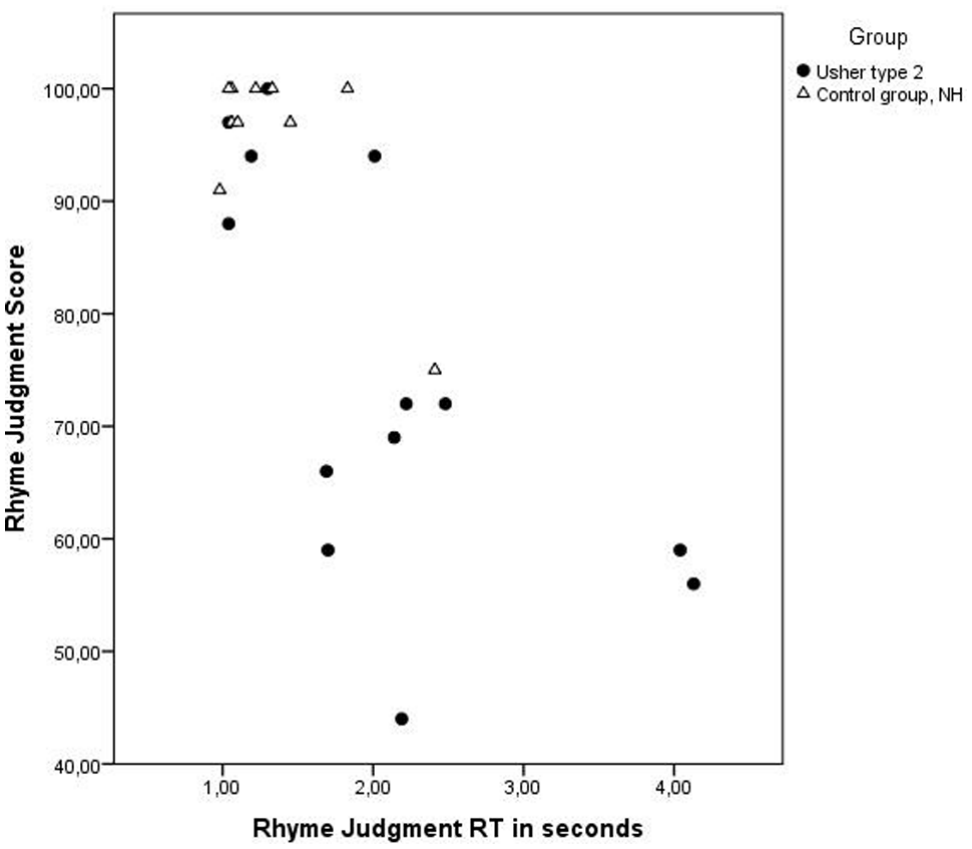
**Displaying RT in seconds and score (% correct answers) for each individual on the test Rhyme Judgment.** Individuals with USH2 are displayed as filled circles and individuals in the control group as triangles. The difference in performance among individuals with USH2 is greater than in the control group with NVH. Performance on this test was affected by degree of visual impairment, but could also be an indication of less stable phonological representations in the group with USH2.

The control group had significantly higher scores, *U*= 114.00, *z* = 3.08, *p* = 0.02, *r* = 0.64 (USH2: *M* = 74.6 and SD = 18.2; NHV: *M* = 95.7 and SD = 7.8), but the difference in RT was not significant, *U*= 34.00, *z* = 1.93, *p* = 0.05 (USH2: *M* = 2.1 and SD = 1.0; NHV: *M* = 1.3 and SD = 0.5) on this test (see **Table 2** and **Figure 3**, for details on performance in the groups). While all participants in the control group had performance above 90%, only four participants with USH2 had performance at or above this score. Regarding the RTs, ten of the participants with USH2 had a RT above 1.5 s, compared to two in the control group.

### Complex Working Memory: Reading Span

The group with NHV had significantly higher performance on this test, *U*= 87.50, *z* = 2.30, *p* = 0.02, *r* = 0.27 (USH2: *M* = 54.6 and SD = 12.8; NHV: *M* = 69.6 and SD = 14.7; see **Table 2** for details on performance in the groups). Eight of the participants with USH2 had scores below 60%, compared to two participants in the control group. Two participants with USH2 were unable to perform this test due to their visual impairment, and were excluded from the analysis of this measure.

### Phonological Working Memory: Serial Recall of Non-Words

**FIGURE 4:**
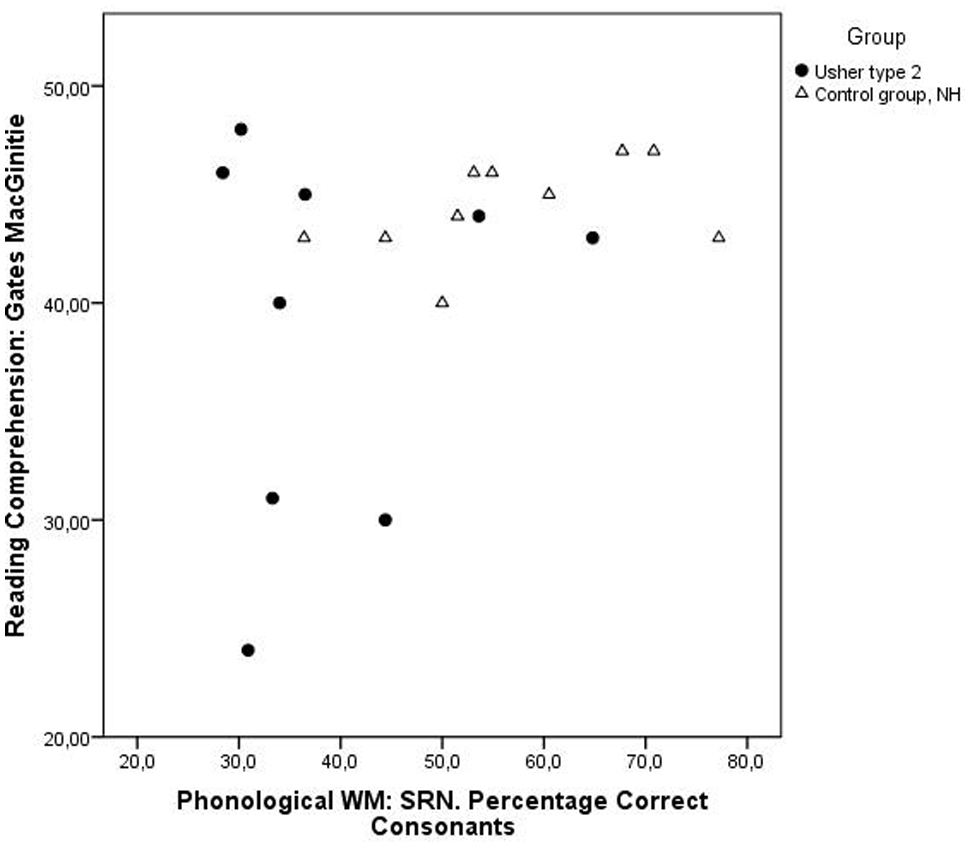
**Displaying performance on phonological working memory (percentage correctly recalled consonants on Serial Recall of Non-words), and score (number of correct answers maximum 42) on reading comprehension.** Individuals with USH2 are displayed as filled circles and individuals in the control group as triangles.

The control group displayed both higher percentage of correct consonants in the recalled non-words *U*= 101.50, *z* = 2.42, *p* = 0.02, *r* = 0.50 (USH2: *M* = 42.1 and SD = 11.9; NHV: *M* = 56.7 and SD = 12.5) and longer span length, *U*= 103.50, *z* = 2.39, *p* = 0.02, *r* = 0.50 (USH2: *M* = 3.9 and SD = 0.9; NHV: *M* = 4.9 and SD = 1.0; see **Table 2**, and **Figure 4**, for details on performance in the groups). Ten of the participants with USH2 had performance at or below 50% consonants correct, compared to two in the control group. Four of the participants with USH2 had a recalled longest sequence at or below four words, while none of the participants in the control group were below this span length.

### Reading Comprehension

There was no significant difference between groups, *U*= 60.00, *z* = 1.24, *p* = 0.22 (USH2: *M* = 39.0 and SD = 8.5; NHV: *M* = 44.4 and SD = 2.2) on this test, but the group with USH2 display higher degree of variability in performance ranging from full score on the test to less than half score (see **Table 2**, and **Figure 4**, for details on performance in the groups). All participants in the control group had a score at or above 40 points (of maximum score 48), while three participants with USH2 had results below this score. Four of the participants with USH2 were unable to perform the test, in two cases because of the visual impairment. In two cases the participants grew too tired during the testing and hence declined participation in the test of reading comprehension.

### Spearmans’ Correlations

There were no significant correlations between age and performance, in terms of score, on the cognitive tests in the group with USH2 (see **Table 3**). There was a significant, moderate correlation between age and performance on complex WM, as well as between age and score on Lexical Matching, in the group with NHV (see **Table 3**). The correlation was negative, indicating that the younger individuals with NHV had higher score on Lexical Matching. In the group with USH2 there were significant, moderate correlations between visual status and RTs on Lexical Matching, Rhyme Judgment, and Physical Matching (see **Table 3**). The correlation between visual status and performance (score) on Physical Matching was significant (see **Table 3**). The correlations between visual status and performance (in terms of proportion correct answers), and visual status and RT on the tests, were not significant (see **Table 3**).

**Table 3:** **Spearman correlations between age, visual status, and cognitive variables**.

	Visual field (best eye)	Visual acuity (best eye)	Age	
	USH2	USH2	USH2	NHV
Age	0.65^∗^	-0.63^∗^		
PTA4 (Left ear)	0.57^∗^	-0.23	0.64^∗^	-0.06
Verbal ability	-0.15	0.02	-0.14	0.26
Reading span	-0.18	0.02	-0.39	-0.75^∗^
Physical matching score	-0.50	0.69^∗∗^	-0.52	0.51
Physical matching RT	0.77^∗∗^	0.77^∗^	0.68^∗^	0.40
Lexical matching score	-0.48	0.19	0.01	0.72^∗^
Lexical matching RT	0.80^∗^	-0.57^∗^	0.81^∗^	0.42
Rhyme judgment Score	0.53	0.25	-0.21	-0.15
Rhyme judgment RT	0.77^∗^	-0.58^∗^	0.66^∗^	-0.01
Reading compr.	-0.54	0.10	0.07	0.09

*^∗^p* ≤ 0.05, ^∗∗^*p* ≤ 0.01.

### Summary of Results

There were significant between-group differences in performance (score) on speeded visual judgment (Physical Matching), phonological processing (Rhyme Judgment), phonological WM (Serial Recall of Non-words), and complex WM (Reading Span). The group with USH2 displayed poorer performance on these measures. There were also significant between-group differences regarding RT on Physical Matching and Lexical access, where the group with USH2 had longer RT. There was no significant difference between groups on reading comprehension. Age and visual decline were moderately correlated in the group with USH2, where increased age was associated with poorer visual performance. Furthermore visual decline and RT on the tests were moderately correlated, such that poorer visual performance was associated with longer RT.

## Discussion

The aim of this study was to examine WM, phonological and lexical skills, and reading comprehension in adults with USH2 in relation to a matched control group with NVH. The general findings were that the group with USH2 had lower performance on complex verbal WM, reduced phonological WM, as well as less accurate phonological processing. Reduced WM and phonological processing was indexed by significantly lower performance and longer RTs on the Reading Span, Rhyme judgment, and Serial Recall of Non-words tests. The effect sizes were moderate to large when the groups differed significantly. However, it is important to note that lower performance was not a general finding in the group with USH2. Several of the participants with USH2 performed comparably to or slightly lower than the control group on the experimental measures. Only a few performed markedly below the control group. An interesting aspect is that performance was varied in participants with USH2 across the different tests such that individual strengths, weaknesses and degree of alertness may have had a stronger influence on performance than their degree of visual impairment, for example. Correlational analysis also indicated that generally low performance was not specifically associated with either higher age or poor vision in the group with USH2. However, two individuals with USH2 displayed generally low performance on all tests, and these cases will be discussed further below.

The variation in performance in the group with USH2 is displayed in **Figures 1–4**, and from this information we can conclude that most participants with USH2 indeed had difficulties on measures of phonological processing and phonological WM; however, some did not.

A slightly unusual finding was the difference in performance on physical matching, a test which is generally used as control measure for general RT. For individuals with NHV the proportion of correct responses is expected to be high. The control group performed at ceiling on this test and had short RTs. Regarding the group with USH2, the majority achieved high scores and had RTs only slightly longer than those of the control group, but four individuals with USH2 displayed low scores and long RTs. Two of these individuals declined participation in the test of complex verbal WM, as well on the test of reading comprehension, because of their low vision. The data on their visual status confirmed both visual field and acuity to be severely limited. Hence, the low performance on physical matching of these two participants was likely an effect of not being able to perceive and/or evaluate the visual stimuli properly. As a group, the participants with USH2 display significantly longer RTs on Lexical Matching and Rhyme Judgment. On both Lexical Matching and Rhyme Judgment the majority of participants with USH2 displayed relatively long RTs, though in the latter case the difference in RT was not significant in the two-sided test of significant difference. A possible explanation is that the participants with USH2 experience visual input to be uncertain due to their visual impairment, and hence have adapted by allowing more time when inspecting visual elements.

Though the finding of significant difference between groups on Physical Matching was unexpected, the differences in performance on tests relying on phonological skills and phonological WM were less so. Even when analyses were run with the two participants with poorest vision excluded from all measures, the pattern of results remained, indicating that phonological processing difficulties are likely to be an issue for persons with USH2. Previous research (i.e., Lyxell et al., 1998; Classon et al., 2013) has investigated the impact of long term hearing loss on phonological skills and found that phonological processing skills decline over time (Rönnberg et al., 2010; Classon et al., 2013). The primary effect of reduced ability to process phonological information, according to Rönnberg et al. (2010) is difficulties when processing speech, and hence speech comprehension can be compromised. However, whether the reduction in phonological skills in adults with long term hearing loss also affects reading comprehension has not been investigated. Most likely this is due to the fact that even though phonological skills are correlated with reading skill in individuals with NH at the beginning stages of reading (Lundberg, 2009; Schaffner and Schiefele, 2013), as the reader becomes more skilled, this correlation becomes less prominent. In USH2, the HI is congenital, and hence could give rise to delayed or divergent development of phonological skills (Wass, 2009; Lederberg et al., 2013; Lyxell et al., 2013; Nakeva von Mentzer et al., 2013) which could have an impact on the development of their reading skill. While there was no significant difference between groups in performance on the test of reading comprehension in this study, three individuals with USH2 performed at or below more than 1 SD of both groups’ means. These three participants also displayed low results on tests of phonological skill, phonological WM, and complex verbal WM. While one of these participants was in the higher end of the age span, the other two were in the middle, and neither of them were among those with poorest vision. Possibly, these participants have not been able to acquire nuanced and stable phonological skills at an early stage due to their HI, and as an effect reading skills later in life are compromised. One of these participants also reported reading to be a very tiring activity, and terminated the test of reading comprehension before the time allotted had expired.

The difficulties with phonological processing experienced by individuals with USH2 in this study could be disruptive for speech comprehension, especially when conversation takes place in noisy environments (e.g., Rudner et al., 2011; Ng et al., 2013). Studies investigating health aspects in persons with hearing loss often find higher levels of fatigue in individuals with hearing loss (Hua et al., 2013).The effort exerted by applying conscious strategies in order to retrieve the information necessary to follow conversations could be one explanation, as suggested by for example Rönnberg et al. (2010). In individuals with deafblindness the access to visual information is also severely limited, hence further increasing the strain on the individual to acquire the information necessary in the conversation. Possibly, the difficulties experienced in extracting information in social situations by persons with USH2 could be part of the explanation behind the findings of Wahlqvist et al. (2013), who found psycho-social health to be significantly lower in the population with USH2, with higher prevalence of headache, fatigue, and depression in comparison to a reference population. Therefore, one of the key goals of rehabilitation should be to help individuals compensate for the loss of information from vision and hearing, and the knowledge gained from studies such as the present could be important in the design of interventions on audiological clinics.

It should be noted that there are inherent challenges in conducting research with populations with deafblindness. Due to the dual sensory loss, and individual variation in degree of loss, it is hard to design a test situation in which all participants with deafblindness would have opportunity to display peak performance. However, none of the participants in the present study reported difficulties with hearing the instructions or test items during the test sessions. All participants were experienced hearing aid users, had their hearing aids checked before the test session, and the FM-systems used during sessions gave further benefit. Compensating for low vision in cognitive testing turned out, not surprisingly, to be a greater challenge. Even though the tests had been adapted for participants with low vision, problems with visibility remained. In particular, the two participants with most advanced RP experienced the tests where stimuli were displayed for only a short time as tiring and had difficulty finding and getting the item in focus before display time for the item expired. As stated, these two participants declined participation in some tests, since they were not able to see the material properly. The impact of the visual impairment on the tests used could be further investigated by including a group with matched visual status, but without HI. Possibly, a group with matching visual impairment would display similar difficulties with fast visual judgment, though performing higher results on the tests of phonological processing skills.

## Conclusion

The performance of the group with USH2 indicated similar problems with phonological processing skills and phonological WM as experienced by other individuals with long-term hearing loss. On tests of phonological processing and phonological WM performance level was significantly lower in the group with USH2 than in the control group with NHV. On the visually displayed tests of phonological processing performance was likely also affected by the problems with visibility, even though with the exception of two participants the individuals in the group with USH2 did not report specific difficulties with visibility. The majority of participants with USH2 had particular difficulties when fast visual judgment was required in combination with phonological processing, such as in the Rhyme Judgment task. However, for several of the measures of phonological processing some individuals performed similar to the control group, whereas a few performed markedly low, despite same level of visual impairment. Information on the level of phonological processing skills could be important in the design of intervention for individuals. Individuals could benefit from extra support and specific training of phonological skills in order to ease communication, thus possibly reducing feelings of stress and/or loneliness. A recommendation for future research would be to further investigate phonological skills in the population with USH2, preferably with separate control groups matched on degree and duration of HI respectively visual impairment. It would also be relevant to study communicative strategies, and to connect these aspects to health and well-being in the group.

## Conflict of Interest Statement

The authors declare that the research was conducted in the absence of any commercial or financial relationships that could be construed as a potential conflict of interest.

## Abbreviations

NHV: normal hearing and vision
P.corr.c: percent correct consonants
USH2: Usher syndrome type 2
WM: working memory
